# Artificial Intelligence for Multiscale Spatial Analysis in Oncology: Current Applications and Future Implications

**DOI:** 10.3390/ijms26168002

**Published:** 2025-08-19

**Authors:** Ali A. Tarhini, Issam El Naqa

**Affiliations:** Moffitt Cancer Center, Tampa, FL 33612, USA

**Keywords:** artificial intelligence, machine learning, deep learning, foundation models, AI agents, multiscale spatial information, radiomics, pathomics, spatial transcriptomics, spatial proteomics

## Abstract

Artificial intelligence (AI) and its machine learning and deep learning algorithms have shown promise in oncological practice. Spatial information analysis in the context of cancer is crucial for its diagnosis and treatment because it can provide an understanding of tumor-microenvironment interactions and reveal insights into response to treatment. AI tools can analyze spatial information at multiple scales, highlighting key disease, clinical, and genetic phenotypes that may reveal underlying mechanisms and molecular markers of response and resistance within the tumor and its microenvironment. By examining tumor interactions at macroscopic (diagnostic imaging) and microscopic (pathology slides and spatial biology) levels, AI can assist in making important diagnostic and prognostic decisions. In this review, we first present an overview of AI and the need for multiscale spatial information in oncology. Then, we examine growing AI applications in the analysis of such information, focusing on diagnostic imaging, digital pathology, and spatial molecular biology. We also discuss applications of large-scale foundation models and task-oriented agentic AI in these fields as emergent technologies. Then, we discuss current limitations for the clinical translation of AI into regular utilization in cancer care and discovery.

## 1. Introduction

AI has captivated the medical world with its unprecedented capabilities to impact diagnostics and treatment in a vast number of ways. In essence, AI can be defined as a technology that can perform tasks that usually require the intelligence of a human [1]. Predictive AI is a type of AI that can make forecasts and predictions based on varying input data resources, including complex spatial patterns, for the diagnosis or prognosis of a patient’s condition. This is primarily characterized by machine learning (ML), a subset of AI that has gained significant popularity; it is an AI technology specializing in the utilization of data analysis to locate patterns and make predictions [1]. ML heavily involves the use of several computer algorithms, including popular neural networks, colloquially termed artificial neural networks (ANNs). These technologies are meant to imitate the neural networks found in the brain; convolutional neural networks (CNNs) are an example of such an ANN that is specialized for detecting spatial patterns in images, which is evident in their use for pattern and abnormality detection in spatial oncology applications [1,2]. Deep learning (DL) is a branch of ML that involves the training of deep neural networks (DNNs) directly from the raw data without the need for hand-crafted features. However, it typically requires larger sample sizes [1,2]. This is useful for spatial oncology applications, where the most relevant patterns are unknown a priori. DL can potentially allow for more extensive analysis of microscopic images, such as pathology slides or spatial biology information, where knowledge is still being developed about the most relevant features in these images. Predictive AI, like those mentioned above, provides scientists and physicians with valuable analytical tools that give insights into patient contexts and future risks.

**Figure 1:**
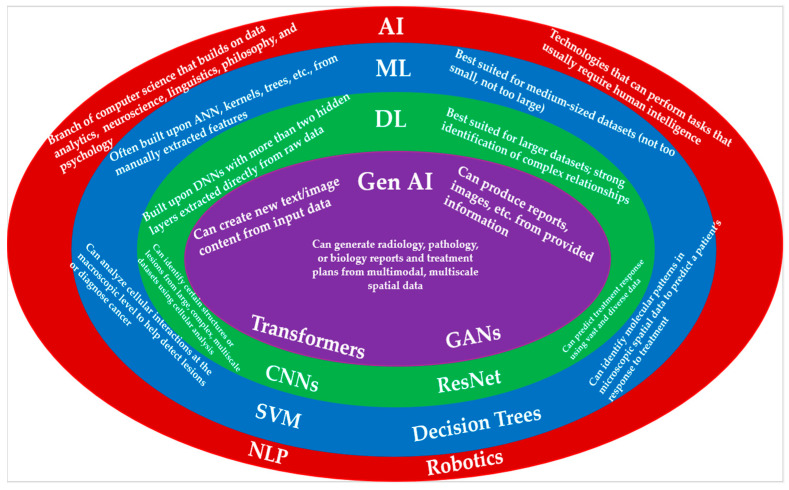
A Venn diagram summarizing the relationships between AI and its subcategories of ML, DL, and Gen AI as they apply to spatial medical data. AI is a highly versatile technology that can be modified and adapted for a wide range of purposes, including radiology, pathology, and spatial biology. While overlapping in certain ways, different branches of AI, such as ML, DL, and Gen AI, each can serve different, unique purposes. The diagram displays descriptions of each type of AI, as well as oncology-related downstream tasks that each can perform using multiscale spatial information. Examples of technologies unique to each type of AI are also included at the bottom of each circle (Abbreviations: NLP: Natural Language Processing; SVM: Support Vector Machine; ResNet: Residual Network; GAN: Generative Adversarial Network).

Another type of AI is generative AI (gen AI), which can create new content, such as images or text, from provided inputs [3]. What separates gen AI from predictive AI is that gen AI generates novel material, rather than making predictions based on the original information. For this reason, gen AI is often used in the context of radiology and pathology report generation, summarizing the findings of oncological analysis at the organ, cellular, and molecular levels. Figure 1 contrasts ML, DL, and Gen AI while also illustrating the relationships between them for spatial oncology studies. Chat GPT and DeepSeek are prime examples of how gen AI has captivated humanity; people have found applications for these technologies in many different aspects of their daily lives [4,5]. The emergence of gen AI in recent years has sparked promise for the future of AI, with predictive and gen AI potentially being utilized together to further optimize treatment planning and clinical decision-making. For instance, predictive AI can analyze images and locate patterns in the data, whereas gen AI can use this information to generate reports or treatment plan templates that physicians can utilize when determining procedures. Overall, AI’s versatility and wide array of possible applications can be used in conjunction with human experts to improve cancer care and overall disease diagnosis and treatment.

AI can also be divided based on scale. Narrow AI, for instance, is specialized only to perform certain tasks, whereas general AI can carry out a large spectrum of different tasks [6]. Most traditional ML and DL algorithms fall under narrow AI. However, general AI has emerged recently as part of the next generation of transformational AI technologies. This rise in general AI is mainly seen in the development of foundation models (FMs), large-scale AI technologies that are trained on very large datasets and can perform many different downstream tasks [7]. These models possess large-scale pretraining abilities and high versatility, displaying strong potential for generalizable and accurate spatial analysis at all scales. Based on whether they make predictions or generate content, FMs can be classified as general predictive or general generative AI. An example of a general generative FM is large language models (LLMs), which specialize in processing and generating human language. What separates FMs from previous technologies is their ability to adapt to different tasks, whether through simple prompting or being fine-tuned into learning these tasks by extended training. These FMs are often trained using self-supervised learning (SSL). This means that labels, which can be scarce and expensive, are not required, as guidance is acquired directly from the dataset itself [7]. Where traditional models require expert labels, FMs can limit the need for the labor-intensive (especially in spatial oncology-related studies where images and slides must be annotated) and expensive work that comes with manually labeled datasets.

The future of medical AI also involves AI agents. This recently introduced technology refers to AI systems that can build on the learnt knowledge of the FM, interact with the environment, and perform tasks autonomously, separating them from traditional technologies that would require humans to trigger an action [8]. AI agents can function alone in a single-agent system, or they can collaborate on specific objectives as part of a multi-agent system. Moreover, agents can further leverage the capabilities of FMs, utilizing them to help break complex tasks down to simpler ones while reasoning effectively [8]. AI agents combine their abilities to interact with the environment autonomously with the large-scale abilities of FMs to carry out tasks with precision and efficiency.

**Figure 2:**
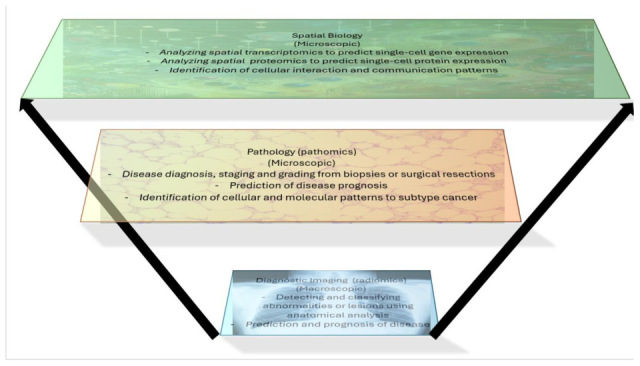
Display of different sources of spatial information organized by scale, with corresponding AI applications. Diagnostic imaging is largely macroscopic, and AI’s applications in its analysis heavily involve diagnostic, segmentation, and prognosis-related tasks. Pathology represents microscopic studies, where AI is often utilized to carry out diagnosis and prognosis at the cellular level. Spatial biology, including spatial transcriptomics and proteomics, involves even smaller-scale examinations, often involving gene expression, cellular interactions, and other microscopic tasks involving high-level molecular analysis.

In the field of oncology, spatial information is important and necessary, whether at the macroscopic or microscopic level. It provides vital multiscale context on the formation of tumors from the molecular, cellular, and tissue levels. This includes an understanding of patient disease status, which can affect the manner in which treatment is developed and administered [9,10]. Specifically, macroscopic images can provide information about tumor locality and size. In contrast, microscopic spatial information can reveal different cellular and molecular compositions, as well as biomarker expression patterns, which assist in the quantification of tumor microenvironments and the overall understanding of a patient’s cancer [9]. Spatial information can come from a variety of sources, with some of the main ones being macroscopic, such as diagnostic imaging, or microscopic, such as pathology slides and spatial biology technologies. Diagnostic imaging can come in the form of different modalities, such as CT scans or MRI images, and is often involved in radiomics (the quantitative analysis of radiology-based images) and radiological practices that aim to diagnose and help treat diseases. Pathology encompasses the study of disease, largely through cell and tissue examination; pathomics is used to quantitatively analyze pathology images. Finally, spatial biology is an emerging field that is used to comprehend cellular and molecular spatial organization, examining how these components interact with one another and the circumstances around them, such as in spatial transcriptomics or proteomics. AI can be incorporated into all of these areas to spearhead enhanced data analysis, leading to improved cancer detection, diagnosis, and treatment. Figure 2 displays different sources of spatial information organized by scale, with potential AI applications for each field.

The different forms of AI all have important roles to play throughout multiscale spatial studies. However, their varying capabilities may render them suited for some forms of spatial analysis over others. This concept will be accentuated by the examples to be discussed in this article. ML, for instance, is often used at a macroscopic level, excelling in anatomical analysis in radiology images. ML-driven technologies are able to distinguish between cell types to identify abnormalities and lesions in large-scale images. That said, in more complex datasets that often involve more macroscopic studies, DL may be the ideal technology to use since ML can be limited to relatively basic datasets in the field of AI. Given that DL is able to analyze larger-scale and more intricate data than traditional ML technologies, DL leverages pattern identification and prediction capabilities of ML models to further delve into cellular and molecular analyses that can help diagnose diseases in pathology and spatial biology-related slides. DL is often used in pathology to locate structures and tissues that may aid in diagnostic decisions, such as identifying tertiary lymphoid structures (TLS) in an immunotherapy patient. That said, FMs may be even more suitable for cellular and molecular analysis in microscopic studies. These models can be trained on greater quantities of data and can digest data that contains complexities and intricacies that even traditional DL algorithms may struggle to work with. Not only that, but since FMs employ general AI, they exhibit a versatility that renders them applicable to wider ranges of tasks. Therefore, FMs can be used for molecular analysis in a wide variety of cancers and can display high prediction and detection accuracy in rarer cancers, as well as common ones, at any scale. This is accomplished due to the enhanced feature extraction ability that FMs have in any type of dataset, even those that are unusually small.

While FMs represent a large portion of the future in spatial oncological studies, they still currently contain reliability and size issues, which will be explored in this article. That said, in the context of spatial oncology, agentic AI can build even further on FMs to enhance spatial analysis. Since AI agents leverage FM capabilities, a multi-agent system with FMs can carry out a wide variety of difficult tasks, such as analyzing cellular interactions to predict gene expression and examining molecular and genetic patterns to identify protein expression trends, which could lead to diagnostic and prognostic predictions. Given the various modes of AI and their differing capabilities, the use of different forms of AI in conjunction can ensure comprehensive and extensive spatial analysis of oncological data at all scales. This approach enables the thorough examination of anatomical, cellular, and molecular interactions from radiology images, pathology slides, and spatial biology information to systematically collect patient information that can, importantly, set the course for successful treatment. The specific ways these different types of AI can be applied to spatial oncology will be evident as examples are discussed.

In this review, we explore the growing applications of AI in multiscale spatial information, focusing on the use of AI tools in diagnostic imaging, digital pathology, and spatial molecular biology. In the writing of this article, we identify and synthesize literature from various sources to provide an overview of cutting-edge technologies with regard to AI in the analysis and use of spatial information. To select examples for this article, an extensive search was conducted on PubMed, arXiv, and bioRxiv databases to identify the current state-of-the-art AI tools in diagnostic imaging, pathology, and spatial biology, focusing on technologies released from 2024 onwards. Focus is placed on traditional AI algorithms of ML and DL, as well as emerging forms of AI in FMs and AI agents. Table 1 compares ML, DL, and FM applications in multiscale spatial oncology analysis from the standpoints of usage, requirements, advantages, and disadvantages.

## 2. AI in Diagnostic Imaging (Radiomics)

Diagnostic imaging with radiomics has played an important role in diagnosing and treating cancer. Adding AI into the fold can further enhance this and raise the standard for performance in radiological practice. This field often involves macroscopic images, where analysis is carried out at an anatomical level. Thus, AI technologies involved in this field focus on locating tumors or masses, for instance, in various tissues and organs. In addition, these tools can look into patterns between structures and tissues to distinguish healthy tissues from abnormal ones at a larger scale. For this reason, ML technologies with extracted computer vision features have often been used in this field where prediction and identification-related tasks are of need at a broad-scale level using moderate to large-scale datasets. ML has been established in AI-radiomics applications as a staple for narrow detection, identification, and prediction tasks as an assistant to radiologists for cancer diagnosis and prognosis. That said, technologies capable of higher-level analysis with more elaborate capabilities have also been introduced to the space to analyze radiological datasets that may contain complexities beyond ML capabilities.

The introduction of DL into diagnostic imaging has expanded the amount of data that can be analyzed by AI. The increased number of computational layers in DNNs allows for the completion of more difficult prediction problems, such as in unstructured datasets. This way, anatomical complexities that require enhanced computational levels can now be analyzed and used to make diagnostic predictions. FMs with general AI expand upon this further; with these models, complex radiological datasets are met with massive, multiplex training datasets that induce high performance in many different downstream tasks. FMs can be used to identify and extract subtle and less-evident features that signify disease progression or even malignancy, as their massive learning capacity allows them to carry out high-level tasks that narrow AI models such as those of ML and DL models are incapable of completing. Moreover, images segmented by FMs have provided radiologists with indispensable framework for accurate and reliable diagnosis. Given the promise these higher-level AI technologies possess, DL, FMs, and even agentic AI technologies more recently have been utilized alongside ML in diagnostic imaging to optimize spatial oncology information analysis at this macroscopic level. With each form of AI capable of serving varying purposes, tools encompassing each of these technologies have played different but valuable roles in the optimization of anatomical analysis using diagnostic imaging.

**Figure 3:**
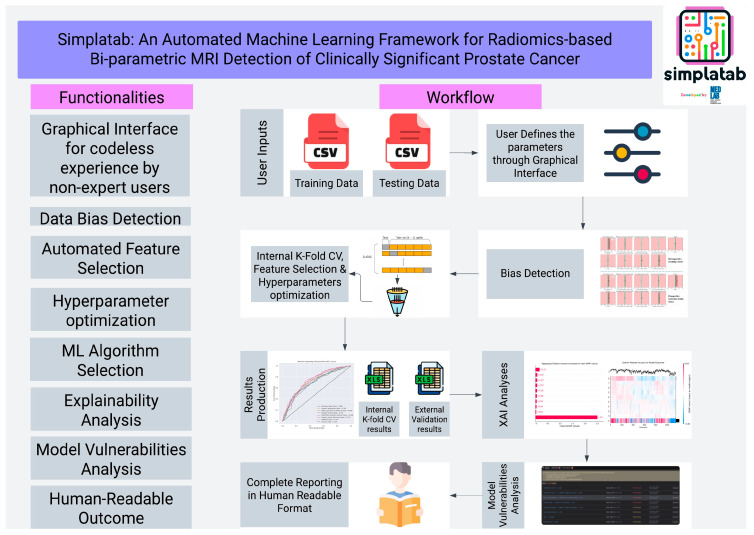
Workflow and downstream tasks of Simplatab. Using training and testing data, Simplatab undergoes a processing pipeline that includes bias detection, internal validation, hyperparameter optimization, feature selection, results production, external validation, and the production of human-interpretable and readable outcomes. These functionalities allow Simplatab to leverage ML capabilities for prostate cancer diagnosis in a manner that humans (physicians/patients) can comprehend [13].

For instance, Simplatab is an ML tool used to address the issue of lesion variability in MRI-based prostate cancer diagnosis [13]. Simplatab utilizes an automated ML framework and is trained on data from 4816 patients for the task of detecting prostate cancer using radiomics [13]. Specifically, its framework integrates feature selection, data bias detection, and model training with explainable analysis, post-training vulnerability detection, and optimization of the hyperparameter, which controls the model’s learning process [13]. Furthermore, Simplatab is open-source, allowing for optimal accessibility. Figure 3 by Zaridis et al. depicts the functionalities of Simplatab, walking through the model’s workflow from input processing to interpretable output production [13]. That said, the tool is limited in the sense that it has yet to be validated in a real-world setting [13]. This raises questions surrounding the ability of Simplatab to perform reliably in unpredictable, real-world settings that occur outside of a controlled lab environment. Without this validation, one cannot be sure that this tool can play a major role in enhancing patient care. To address this, validation of Simplatab on real-world datasets beyond clinical trials is necessary to evaluate its effectiveness in uncontrolled circumstances. While Simplatab does show promise overall, it is ultimately limited by the fact that ML technologies do not perform well with complex, unstructured datasets. For this reason, DL tools have been increasingly utilized in diagnostic imaging to analyze this more intricate data. One such DL tool is RE-ViT. This model is a vision transformer, a type of DL model architecture encompassing the transformer attention concept of being able to point out and extract the most relevant information within a dataset, an ability that most ML technologies do not possess [14]. The model can leverage the transformer architecture also to process visual data, hence why it is a vision transformer. Not only that, but the model is embedded with radiomics, increasing interpretability and performance in analyzing macroscopic tissue and organ structures [14]. This integration occurred through early fusion, where images are split into patches and are embedded with extracted radiomics features [14]. For the task of medical image classification on a dataset of 830 images from 600 female patients, RE-ViT achieved an area under the receiver operating characteristic curve (AUC) of 0.950 ± 0.011, displaying stronger performance than compared models, with the second-highest AUC being 0.848 ± 0.027 [14]. However, RE-ViT has some limitations; for instance, validation on large-scale datasets is needed to evaluate the model’s strength and generalizability, as datasets used were confined to public benchmarks that do not completely reflect real-world circumstances [14]. This not only raises concerns about its performance in a non-controlled setting, but generalizability is also limited by the fact that the model is only utilizing public benchmark datasets. In order to solve these issues, dataset expansion should be explored to test the model’s performance in a real-world setting, as well as to improve its generalization capabilities. Nonetheless, RE-ViT’s image classification abilities warrant great promise. DL has shown strong versatility in diagnostic imaging, with another promising radiomics-driven DL tool being SALSA. This technology is trained on 1598 computed tomography (CT) scans and 4908 liver tumors and is applied for downstream tasks of volume quantification and tumor identification, which require a strong understanding of anatomical structures and their interactions [15]. Patient-wise detection precision is a strength of this tool, as it posted a precision of 99.65% for this aspect; additionally, SALSA displayed a dice similarity coefficient (DSC) of 0.760, showcasing strong overlap [15]. Despite this, the tool comes with limitations; while experts in radiology annotated the training dataset, only one rater carried out labelling per CT scan, which may cause biases to emerge from the labels. This puts model outputs into question while raising concerns about its reliability in performance. This issue can be mitigated, though, through the use of consensus-based annotations [15].

Despite the promise of DL technologies, their training datasets are often specific and limited, narrowing their downstream task capabilities. FMs with general AI, on the other hand, have brought an enhanced aspect to diagnostic imaging and radiomics through their training on massive, diverse datasets and the ability to be fine-tuned via supervised learning for specific tasks after pretraining, which requires much less training effort than building a DL model from scratch, for example. MedSAM, for instance, is a general predictive FM that is developed for the segmentation of medical images. MedSAM is trained on a vast dataset including over 30 cancer types and 10 imaging modalities [16]. A study fine-tuned this model for cervical disc segmentation and carried out feature extraction for 924 radiomics features from each disc segmented in T1 and T2 MRIs [17]. This fine-tuned MedSAM achieved an average DSC of 0.93, underscoring noteworthy performance for segmentation of cervical discs [17]. This way, tumor localization, organ boundary determination, and even pre-surgical planning can be optimized, setting radiologists up for enhanced diagnostic and prognostic success, as well as increased success in eventual treatment plans. Mishaps in segmentation can lead to misdiagnoses and lackluster treatment plans, which is why MedSAM’s performance displays promise. Through its large-scale pretraining and task-specific fine-tuning, the model underscores the potential of FMs to serve as reliable image segmentors that lay the foundations for accurate cancer diagnosis and treatment. Limitations to this study, however, include the fact that it was retrospective, resulting in a lack of included clinical data, which may have altered performance, as the patient population may not be properly represented [17]. This means that once the model comes across patient data, it may not be adequately prepared to accurately perform on it. To address this issue, prospective studies that include representative clinical data may be crucial to improving direct performance on clinical data. Despite this, the segmentation abilities of MedSAM enable enhanced anatomical analysis, allowing researchers to better differentiate between healthy and cancerous tissue, which can lead to thorough investigations of disease. FMs have displayed potential in diagnostic imaging beyond image segmentation, however. For instance, another FM in this field was developed by Pai et al. This general predictive model employs SSL, utilizing a dataset of 11,467 radiographic lesions via CT scans from 2312 patients, and is developed for cancer imaging biomarker discovery [18]. In anatomical site prediction, the model posted a mean average precision (mAP) of 0.857 (95% CI 0.828–0.886) after being fine-tuned on a dataset containing 3830 lesions, outperforming all compared baseline methods [18]. Given the amount of anatomical analysis carried out in diagnostic imaging, this performance is both promising and valuable. That said, this model is limited by its lack of transparency, making it difficult to interpret how the model reached or produced certain outputs [18]. Physicians’ trust in AI is crucial to widespread clinical adoption, so research into how this model works may be important for its future clinical use.

One of the most promising aspects of FMs is their ability to perform multiple tasks at a high level. Such an FM impacting diagnostic imaging and radiology is RadFM. RadFM is a general generative model trained on a large dataset spanning 17 medical systems and over 5000 diseases [19]. The model is able to generate reports, diagnose diseases, recognize modalities, and perform other tasks. RadFM performed highly on several tasks; for instance, for the task of modality recognition, the model posted an accuracy score (ACC) of 92.95%, significantly greater than the next highest score out of a comparison group of other FMs, which was 84.25% [19]. This underscores the model’s ability to provide radiologists with important information about the nature of the data being examined, which can guide treatment planning and result interpretation. While RadFM displays strong performance, it is limited by the small number of 3D images in its training dataset, which is accentuated by missing metadata in these 3D images, leading to features such as imaging spacing being lost, which can impact the determination of tumor size [19]. This information is needed for diagnosis and treatment planning, so the addition of 3D images with sufficient metadata into the model’s dataset is important for ensuring RadFM’s dependability and accuracy in macroscopic, anatomical analysis. Nonetheless, RadFM displays strong potential as an FM in the diagnostic imaging space. Additionally, agentic AI is showing promise in diagnostic imaging, leveraging the abilities of FMs to achieve even higher performance. For instance, mAIstro is an open-source multi-agent framework for automated end-to-end development of radiomics and deep learning models in the field of medical imaging [20]. This system is capable of segmenting images, extracting radiomic features, and carrying out exploratory data analysis. These agents provide autonomy to LLMs and enhance their abilities by interacting with the environment surrounding them. By giving LLMs the ability to reason, mAIstro and similar agentic systems represent a new frontier for expanded radiomics-based research, providing for the enhancement of diagnostic imaging beyond current standards [20]. In this multi-agent system, a single agent is responsible for one of the following tasks: radiomic feature extraction, feature selection, exploratory data analysis, segmentation, classification, regression, and other imaging-related tasks [20]. Spatial analysis of diagnostic images is enhanced by this system, where a plethora of tasks can be simultaneously, efficiently, and accurately addressed. While mAIstro is largely promising, it is limited by a reliance on the capabilities of the LLMs that it leverages, which are not always reliable [20]. However, this system shows great potential for the future of AI in diagnostic imaging and radiomics, as well as a macroscopic analyzer of tissue and organ interactions. Overall, many recent advancements have been made in this field, providing optimism for future AI applications in the pursuit of enhancing disease diagnosis in imaging.

## 3. AI in Pathology (Pathomics)

Pathology is essential to cancer diagnosis and treatment guidance and has proven to be a valuable source of microscopic spatial information, contributing greatly to advancing research in this area. As AI has been integrated into this field, the analysis of spatial information has heightened. By nature, pathology and pathomics are often characterized by microscopic analysis; since biological samples are often examined on pathology slides via microscopes, where specimens are examined at cellular and even molecular levels to locate discrepancies, there is a requirement for analysis of cell–cell interactions and characteristics that is mainly feasible at a microscopic level. Therefore, AI tools that are utilized in this field must be capable of analyzing microscopic images with high accuracy. With the popularization of digital whole-slide images (WSIs), AI has played an increased role in pathomics and the diagnosis of disease using microscopic spatial analysis. That said, given the complexity of the analysis in this field, ML algorithms are not as widely used for pathomics analysis. Rather, higher-level DL algorithms have become a widespread AI tool in this field for their ability to digest cellular and molecular data with greater success.

Regarding narrow, specific tasks, such as identifying certain cellular structures or locating molecular patterns, DL has displayed great promise. However, where labels have been scarce or where an AI tool generalizes for multiple tasks, FMs have been increasingly utilized. These models are not only capable of performing narrow tasks at a high level due to large-scale pretraining and objective-specific fine-tuning, but they are also able to perform a multitude of varying tasks without a drop in performance. Therefore, FMs have been used to carry out cellular and molecular analysis of pathology slides to diagnose disease, classify structures, make patient-related predictions, and perform other tasks via microscopic studies. While FMs with general AI have shown immense potential for the future of pathological analysis, DL tools remain a mainstay regarding AI applications in pathology for their high-level accuracy in narrow, specific tasks.

**Figure 4:**
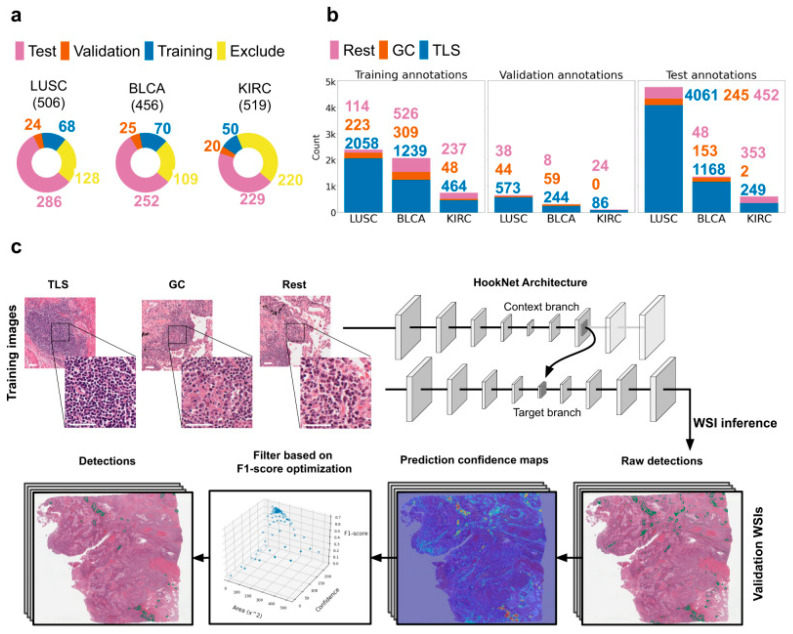
Data pipeline and model architecture of HookNet-TLS. (**a**) Slides from TCGA are initially separated into groups for training, validation, and testing. (**b**) Slides are extensively annotated for TLS, germinal centers (GCs), and lymph nodes, along with other tissue types. (**c**) HookNet-TLS has a complex model architecture consisting of multi-resolution patch-based training, as well as steps such as object detection enhancement and WSI inference, which occur after data processing [21].

For example, HookNet-TLS is a DL tool used for the quantification of tertiary lymphoid structures (TLS) and the identification of germinal centers in hematoxylin and eosin (H&E)-stained pathology slides by examining cellular patterns and interactions [21]. TLSs are accumulations of immune cells that form due to injury or inflammation and have been shown to be key indicators of response to cancer immunotherapy, which is why the abilities of HookNet-TLS show great potential. The model is developed on 1019 slides from the Cancer Genome Atlas (TCGA), including varying cancers such as lung squamous cell carcinoma, muscle-invasive bladder cancer, and clear cell renal carcinoma [21]. Figure 4 by van Rijthoven et al. illustrates the data pipeline used for HookNet-TLS, as well as the model’s architecture [21]. HookNet-TLS aims to address the issue of the difficulty in manually recognizing TLS in H&E; however, the tool was extensively labeled by trained researchers, which may have been labor-intensive and time-consuming [21]. To address this obstacle, FMs using SSL, such as UNI, have been developed. This general predictive model is trained on over 100,000 diagnostic H&E-stained WSIs, which account for over 100 million images [22]. UNI is able to carry out vital tasks such as disease subtyping, tissue classification, and disease diagnosis, which not only provide important guidance for pathologists but can also spearhead diagnostic decisions via microscopic analysis [22].

The use of general AI to perform a wide array of tasks, as evident with UNI, has led to an increasingly widespread interest in FMs throughout the field of pathology for use in different areas. For instance, another FM that has captivated pathologists is Prov-GigaPath, excelling in cancer subtyping and mutation prediction. This general predictive FM is trained on 1.3 billion 256 × 256 pathology image tiles spanning 31 major tissue types and 30,000 patients [23]. The model is pretrained using Prov-Path, a large pathology dataset, with training continued via GigaPath, a two-stage transformer that is able to represent a slide as a succession of “tokens” [23]. The model can differentiate between varying types of cells, possessing the capability to recognize cell-level biological patterns that could differentiate some cells from others. Such analysis opens the door for an accurate cancer diagnosis, which raises the potential for successful treatment plans. For the task of cancer subtyping, Prov-GigaPath achieved an average AUC of 0.903, and for lung adenocarcinoma five-gene mutation prediction, it posted an average AUC of 0.708 [23]. Both scores outperformed comparative groups of other FMs. One limitation of this tool is the significant discrepancy between its AUC for cancer subtyping and that of mutation prediction, which could hinder the model from achieving true generalization [23]. In order to address this obstacle, data can be added to the model’s dataset, or task-specific fine-tuning can be carried out to raise the performance of mutation prediction. Nonetheless, Prov-GigaPath’s ability to subtype cancer and predict mutations remains promising as an innovative technology in the analysis of spatial information in pathology. Pathology FMs have also been developed for gene expression-related tasks. This is evident in OmiCLIP, another FM utilizing spatial information from pathological slides. This general predictive model is trained on 2.2 million paired H&E-stained tissue images and transcriptomic data that covers 32 organs [24]. The model’s capabilities include annotation using marker genes or bulk RNA sequencing, spatial transcriptomics gene expression prediction, and tissue alignment, valuable molecular and cellular analyses that can lead to cancer diagnosis and prognosis [24]. Through such microscopic analysis, valuable information about patients on a gene-expression level is gathered and can be used when planning for treatment and predicting prognosis. However, a main hindrance of this model is that zero-shot performance, where performance is evaluated without any sort of fine-tuning, is limited by the size of the training dataset [24]. That said, this limitation can potentially be solved by an increase in diversity in the model’s dataset. Overall, as a source of spatial information, pathology-related slide analysis with AI has led to major advances in identification and prediction related to the study and diagnosis of cancer at the cellular and molecular levels. Future advances in FMs and agentic AI look to expand the current impact of AI models through enhanced predictive abilities and increased efficiency in performing tasks.

## 4. AI in Spatial Omics Biology

Spatial biology is an emerging contributor to microscopic spatial information and may provide vital context for complex molecular mechanisms that underlie biological profiles and patient conditions. As AI has been incorporated into this field, spatial biology analysis and applications have progressed rapidly. Spatial biology examines microscopic functions at a true molecular level, encompassing molecular interactions at the gene and protein levels. At this stage, intricate analysis is carried out to truly gain an understanding of tumor composition and interaction with its microenvironment. Given the complexity of cancer as a disease, it is highly important to explore these molecular features, as understanding a tumor’s gene and protein expression can lay the foundations for major diagnostic and prognostic decisions, as well as future treatment plans. However, since spatial biology analysis involves intricate examinations at the molecular level, AI tools in this field must be able to digest complex microscopic data, identify structures, and locate patterns between cells and molecules. Therefore, many of the technologies found in spatial biology analysis are FMs, with agentic AI also beginning to emerge as a promising tool. These tools are equipped to handle vast, complex datasets that provide the understanding of spatial biology needed to carry out high-level molecular analysis. Overall, the complexities present in spatial biology analysis require AI-driven tools that can identify and analyze complex molecular structures and interactions. The tools utilized in this field deal with microscopic proteins, genes, and other molecular entities, leveraging them to gain an understanding of tumor composition and behavior that leads to successful diagnoses and eventual treatment.

**Figure 5:**
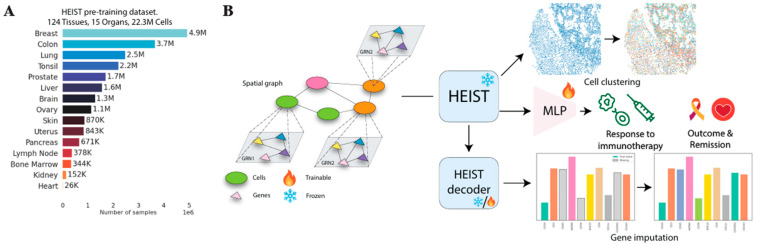
Pretraining and downstream tasks of HEIST. (**A**) HEIST is trained on a vast dataset consisting of various ST data sources spanning 124 tissues, 15 organs, and 22.3 million cells. (**B**) The model is able to create a “spatial graph,” which allows it to carry out tasks such as immunotherapy response prediction, cell clustering, and gene imputation [26].

Spotiphy, for instance, is an AI-driven computational tool that converts sequencing-based spatial transcriptomics data into single-cell-resolved whole-transcriptome images [25]. Whole-transcriptomic profiling is very important to gain an understanding of how tumors interact and function within their environment, and Spotiphy achieves this using gen AI through the generation of single-cell RNA expression profiles from all cells examined [25]. This molecular-level analysis can greatly contribute to disease prognosis, as a better understanding of tumor behavior is achieved, providing a framework for personalized treatment plans based on the expression profiles of the patient’s tumor. However, the tool can be limited by a dependence on input data quality for high-level results, accentuating the issue of data quality that hinders many current AI models [25]. Nonetheless, Spotiphy’s computation-driven whole-transcriptomic profiling is important for the enhancement of precision oncology through further understanding of a patient’s tumors. As discussed, such a level of molecular analysis requires high computational power and learning capacity, which is why the use of FMs has increased in spatial biology. For example, HEIST is a graph FM for spatial proteomics and transcriptomics data. HEIST is trained on a dataset containing over 22.3 million cells from 124 tissues covering 15 organs; the technology is able to model spatial interactions between cells while allowing for information exchange between genes and cells in a cross-modal manner [26]. This allows researchers to visualize how different cell types, such as tumor and immune cells, interact within the tumor microenvironment and how cancer cells are organized. This information can then be used to develop treatment plans that target certain cell populations, identify tumor regions of the greatest threat, and formulate procedures to eliminate cancer cells with maximum accuracy and efficiency. Figure 5 by Madhu et al. provides an overview of the framework of HEIST, examining its pretraining dataset and downstream task capabilities [26]. In addition, HEIST is capable of clinical outcome prediction, gene imputation, cell clustering, and cell type annotation, underscoring the advantages of general AI models in performing a vast number of valuable diagnostic and treatment-related tasks. To exemplify the model’s high performance, for the task of cell type annotation on a UPMC neck dataset, HEIST improved on other FMs by 311.3%, showing a promising ability to analyze spatial biology-based information [26]. However, HEIST has limitations, including the requirement for high computational power and resources for inference applications, which can be energy-consuming and inefficient [26]. Nonetheless, the potential HEIST displays in the utilization of spatial transcriptomics and proteomics are promising.

While HEIST is capable of utilizing both spatial transcriptomics and proteomics data, VirTues is an FM for spatial biology that specifically utilizes spatial proteomics. The model is trained on a diverse dataset covering melanoma, breast, and lung cancer tissues and handles tasks relating to biological discovery, clinical diagnostics, and retrieval tasks relating to patients [27]. Regarding the cells that denote the molecular profile of tissues, VirTues is able to distinguish between the composition of these cells and their spatial distribution, an important capability for understanding spatial context regarding cellular and molecular interactions within the tumor and its microenvironment [27]. Without this impactful information, it may be difficult to understand how the tumor is behaving from a cellular standpoint, which can make it difficult to determine the likelihood of the tumor metastasizing or posing a significant threat. Therefore, the model’s capabilities underscore a vital form of analysis in the field of spatial biology. VirTues’ high performance is exemplified by its accuracy of 0.85 in binary classification in suppressed expansion regions, improving on a compared model by 4.11% [27]. That said, additional validation is needed on more vast and diverse datasets to ensure VirTues is scalable, which may be required before its widespread adoption [27]. In essence, analysis using spatial proteomics can provide valuable cellular and molecular context regarding the nature of the tumor microenvironment. KRONOS is another FM built for spatial proteomics. This mainly general predictive FM is trained on 47 million image patches spanning 16 tissue types, 175 protein markers, and 8 fluorescence-based imaging platforms [28]. KRONOS performs downstream tasks such as treatment response prediction, cell phenotyping, and retrieval. Not only can the model distinguish between cells, which can be applied to stratifying between cancer cells and healthy cells, but it can also predict response to treatment, which can assist physicians in determining optimal treatment procedures. On cell phenotyping, KRONOS achieved an accuracy of 0.7358 ± 0.0089, with the next highest score out of a comparison group of other FMs being 0.6210 ± 0.0121, displaying the strong performance of KRONOS for potentially differentiating between healthy and cancerous cells and tissues [28]. However, the model’s performance can be improved by the addition of ion-based modalities to its training dataset, such as mass cytometry images, since these can provide information about the genetic content, number, and size of cells that could allow cell phenotypes to become more apparent [28].

As an emerging technology building off the vast abilities of FMs to even further analyze spatial biology information, agentic AI has also been applied in this field. SpatialAgent is an LLM agent that leverages FM capabilities to carry out autonomous reasoning, completing tasks such as hypothesis generation, multimodal data analysis, and experiment design via spatial biology information [29]. The model employs two modes for operation: its co-pilot mode is designed for interaction and allows individuals to make changes to task definitions as the agent operates, while its autonomous mode carries out objectives entirely without human intervention or interaction. In the identification of biological variance and spatial organization using cellular and molecular analysis, SpatialAgent improves upon other current computational methods by 6.0–19.1% [29]. As highlighted throughout this article, this determination of spatial organization is of immense importance because by identifying where different cells are organized and how they interact at a microscopic level, researchers can determine where the tumor resides, as well as how quickly it may be dividing, how likely the tumor can be contained, and other indispensable features that set physicians up for prosperous treatment plans. However, by relying on LLMs, as evident with other emerging agentic AI technologies, agent performance may be limited by faults in the FMs that are leveraged, which is why there needs to be consistent validation and monitoring of FMs to limit the misinformation that may be spread [29]. Nonetheless, agentic AI with FMs represents a large portion of the future of spatial biology utilization and analysis.

## 5. Discussion

While AI holds great promise for impacting spatial information analysis, several limitations come with the technology that may hinder its widespread integration, especially in spatial biology, where datasets can be highly complex and diverse. One issue with the use of AI in spatial studies is generalizability. Specifically, if a model is trained on a dataset from one spatial area, it may be difficult to utilize that model in other areas as-is [30,31]. Given how complex certain tumor compositions and microenvironments can be, each with unique, intricate patterns, it is challenging to develop an AI model that can analyze a wide range of tumors, each originating from varying circumstances. Privacy issues also come with the use of AI in this space; algorithms may inadvertently leak sensitive information that can put individuals at risk. It is important that algorithms keep patient information gathered from spatial studies confidential. This is especially the case with many of the diagnostic images and genetic information that AI tools may encounter during analysis. Therefore, consistent validation of models even after deployment is necessary to ensure that patient privacy is maintained [30,32]. Privacy can also be compromised by the hacking of AI algorithms or other forms of adversarial attacks, which risk the release and disclosure of this sensitive, patient-specific information, which could lower model performance in the aspect of security and user trust [33,34,35,36,37]. Other data-related issues are also noteworthy for AI’s application to spatial studies. Consistently high performance by AI algorithms is often contingent on high-quality data; if a dataset lacks organization or quality, the output produced by a model is likely to mirror such a caliber [38]. Low-quality data that contains confusion and inconsistencies can potentially mislead models, leading to false and unreliable outputs as a result of faulty input data that fails to achieve intended goals. Not only that, but data of an insufficient caliber can make certain patterns and structures less apparent, which can also decrease model performance. In essence, for AI algorithms to reach their full potential, they should be provided with high-quality, clean datasets that facilitate higher performance in models by reducing the difficulty of interpreting and analyzing data. This, however, can be challenging in fields such as spatial biology; the complexity and diversity that spatial biology datasets can employ may hinder the consistent construction of high-level datasets. That said, this issue can be present in all sources of spatial information where intricacy can sometimes get in the way of high-caliber dataset assembly. Moreover, many ML and DL algorithms utilize labeled datasets, where labels can be expensive or labor-intensive. FMs and SSL are helping to mitigate this issue; however, they limit the use and need for labels when analyzing datasets. That said, the price of AI software can still be costly [39].

Innovative technologies such as FMs employing general AI are highly promising and representative of future AI applications in spatial studies. However, these large-scale models can be limited by their size. FMs often have billions of parameters, which correlate to a model’s learning capabilities, while a traditional neural network may have thousands or millions. This means that more graphical processing units (GPUs) need to be trained, which in turn leads to the use of greater amounts of computational power [11]. This increased requirement for energy results in higher carbon emissions, which can have an adverse environmental impact. Greater computational needs can also impact the economy, with public health costs seeing tenfold increases if energy is released at backup generators’ maximum levels [11]. These computational demands can be accentuated by oncological spatial information analysis, as the cellular and molecular analysis carried out by AI tools in this field, especially when studying molecular data such as spatial transcriptomics and proteomics, is rarely straightforward and requires immense pre-training and heavy analysis. The data of this multi-dimensionality demands greater computational power, which may result in increased economic and environmental consequences. In addition, FMs, especially models that directly digest and produce human language like LLMs, can produce false outputs known as “hallucinations” [12]. Such outputs can be harmful to individuals who may make detrimental decisions based on faulty information from these FMs/LLMs. However, entropy-based estimators are being developed that can quantify when an LLM is likely to “hallucinate,” allowing for action to be taken to prevent negative societal impacts from these outputs [12]. Similar approaches may need to be developed when dealing with FM techniques. General AI with FMs also poses the risk of bias, where datasets may fail to account for certain groups or parts of information, which, in the case of AI in spatial studies, can limit performance and generalizability [40]. Despite this, the issue can be addressed through consistent model validation and the development of more comprehensive and expansive datasets that cover a wider range of information [40].

AI agents are highly promising in science as a whole and can raise the standard for precision and efficiency in research through the combination of FM capabilities and the agentic ability to interact with the environment autonomously. A recent innovation, the model context protocol (MCP), looks to increase efficiency even beyond that of AI agents alone. AI systems can sometimes experience data isolation, lacking the information required to perform at a high level. MCP addresses this by standardizing the way AI models connect to data sources, providing a baseline for communication between FMs and agents, for instance [41]. Through MCP, efficiency increases even further as FMs and AI agents can exchange information via MCP to facilitate individual tasks and achieve broader goals with enhanced precision and timeliness [41]. The communication standardization provided by MCP underscores the promising ability for agentic AI and FMs to spearhead great impacts in the analysis of spatial studies. Not only does efficiency increase with MCP, but it also facilitates cross-technology communication, enabling more in-depth analysis of molecular and cellular interactions at both microscopic and macroscopic levels that could increase confidence in diagnostic-related decisions. That said, for agents to reach their full potential, they must overcome certain limitations; as discussed, agents can sometimes depend largely on FMs such as LLMs, which do not always ensure high-level or reliable performance. Furthermore, missing information in datasets can result in lackluster agent performance [42]. Nonetheless, agentic AI shows the potential to enhance many aspects of spatial studies, using spatial information to potentially improve disease diagnosis, disease prognosis, treatment response prediction, and other vital tasks. AI agents, FMs, and MCPs can play a valuable role in future spatial information analysis.

## 6. Conclusions

Overall, AI has proven to have a wide array of applications in the use of multiscale spatial information, which is vital in oncology practice due to the understanding and context its analysis provides about the nature and interactions of tumors from the macroscopic anatomical level to the microscopic cellular and molecular levels. The main sources of spatial information are diagnostic imaging at the macroscopic level and pathology and spatial biology at the microscopic level, which have proven to contain numerous applications for AI technologies. ML and DL technologies under predictive AI have used this information to enhance inference and forecasting abilities through extensive multiscale analysis. Moreover, Gen AI has been able to generate reports, plans, and treatment components that can optimize treatment development and delivery. In the context of spatial information analysis, the technology has been able to leverage molecular analysis for the generation of gene expression profiles and can interpret diagnoses based on anatomical and cellular analysis of diagnostic imaging and pathology slides to propose treatment procedures. FMs expand on these technologies by digesting vast, diverse datasets and performing several different tasks. These models have expanded upon predictive AI abilities as well as those of Gen AI to raise the standard for spatial information analysis and represent much of what the future holds for AI in these fields. In the case of pathology slides and spatial biology information, where complex molecular analysis is carried out on unique, intricate molecular interactions, the large-scale pretraining and task-specific fine-tuning of FMs allows for in-depth analysis of these microscopic areas. Not only that, but agentic AI has emerged as a promising tool for spatial information analysis, utilizing task-specific agents to perform a plethora of different downstream tasks that can impact cancer care in many ways. These technologies can truly make the most of valuable spatial information and demonstrate what the future may hold as these technologies continue to expand their applications. For instance, a multi-agent system connected via MCP could potentially carry out spatial organization modelling, cellular or molecular analysis, diagnostic predictions, and treatment plan generation all simultaneously, building off the enhanced analysis capabilities of FMs. That said, limitations need to be overcome for these tools to reach their full potential, such as ethical concerns, data quality issues, potential false outputs, the risk of hacked algorithms, etc., but the promise shown by these technologies is still highly noteworthy. In the future, research could aim to address many of these limitations. For instance, studies could investigate how to further integrate multi-omics data, such as spatial transcriptomics, proteomics, and even metabolomics, to develop stronger contextual understandings for tumor composition and behavior that can lead to enhanced spatial analysis. Moreover, research could be carried out for greater generalization of current AI models, looking into how greater quantities and a greater quality of data can be conjoined to cover greater amounts of cancer cases that allow models to perform effectively on a wide array of cancer types. Focusing on physician adoption of AI tools, future directions could include an increase in the level of understanding of how AI models produce their outputs, which could raise physician trust and the willingness to incorporate these tools into daily clinical use. The future of spatial information analysis heavily involves the use of AI, which has transformative capabilities in this field. Table 2 summarizes the examples discussed throughout this article in each of the sources of spatial information mentioned.

## Figures and Tables

**Table 1:** Comparison of machine learning, deep learning, and foundation model applications in multiscale spatial oncology analysis.

Type of AI	Machine Learning [1,2]	Deep Learning [1,2]	Foundation Models [7,11,12]
Usage	Mainly macroscopic, less complex datasets, such as those of diagnostic imaging, and relies on handcrafted features	Macroscopic and microscopic datasets that are of greater complexity, often dealing with complex visual patterns that are not available through handcrafted features	Macroscopic and microscopic datasets of very large quantities and intricacies that can be promoted or fine-tuned to specific tasks in diagnostic imaging, pathology, or spatial biology
Requirements	Labelled datasets with extracted features that have limited complexities; often radiological	Could be labelled or partially labeled; works on the raw input data	Labelled or unlabeled dataset, depending on the task; greater amount of computational power than ML and DL; can be in any of the spatial information sources
Advantages	Task-specific, limited size sample requirement; strong prediction and inference abilities via anatomical on datasets with limited intricacy	No need for feature extraction or selection; high-level anatomical and cellular analysis on complex structured and unstructured data	Can extract features (embeddings), make predictions, and generate content from highly complex and vast datasets that deal with deeply multiplex molecular structures
Disadvantages	Limited predictive power; can struggle to interpret complexities within data such as cellular and molecular entities	May not be able to analyze heterogenous abnormalities or diverse datasets with unique tumor interactions	Requires high levels of computational power and may be subject to producing false outputs (hallucinations)

**Table 2:** Summary of AI tools for multiscale spatial analysis.

AI Tool	Type of AI	Downstream Tasks	Scale
Simplatab [13]	Traditional Machine Learning	Prostate cancer diagnosis	Macroscopic (Diagnostic Imaging)
RE-ViT [14]	Deep Learning	Medical image classification	Macroscopic (Diagnostic Imaging)
SALSA [15]	Deep Learning	Volume quantification and tumor identification	Macroscopic (Diagnostic Imaging)
MedSAM [16,17]	Foundation Model	Medical image segmentation	Macroscopic (Diagnostic Imaging)
Pai et al.-developed model [18]	Foundation Model	Cancer imaging and biomarker discovery	Macroscopic (Diagnostic Imaging)
RadFM [19]	Foundation Model	Report generation, disease diagnosis, modality recognition	Macroscopic (Diagnostic Imaging)
mAIstro [20]	Agentic AI Technology	Image segmentation, radiomic feature extraction, exploratory data analysis	Macroscopic (Diagnostic Imaging)
HookNet-TLS [21]	Deep Learning	Tertiary lymphoid structure identification	Microscopic (Pathology)
UNI [22]	Foundation Model	Disease subtyping, tissue classification, disease diagnosis	Microscopic (Pathology)
Prov-GigaPath [23]	Foundation Model	Cancer subtyping, mutation prediction	Microscopic (Pathology)
OmiCLIP [24]	Foundation Model	Tissue alignment, spatial transcriptomics, gene expression prediction	Microscopic (Pathology)
Spotiphy [25]	Machine Learning	Whole-transcriptomic profiling	Microscopic (Spatial Biology)
HEIST [26]	Foundation Model	Clinical outcome prediction, gene imputation, cell clustering	Microscopic (Spatial Biology)
VirTues [27]	Foundation Model	Clinical diagnosis, biological discovery	Microscopic (Spatial Biology)
KRONOS [28]	Foundation Model	Treatment response prediction, cell phenotyping	Microscopic (Spatial Biology)
SpatialAgent [29]	Agentic AI Technology	Hypothesis generation, multimodal data analysis, experimental design	Microscopic (Spatial Biology)
